# Clinical features of oral and oropharyngeal squamous cell carcinoma HPV related

**DOI:** 10.1186/1753-6561-8-S4-P49

**Published:** 2014-10-01

**Authors:** Pedro Leite Azevedo, Priscila Marinho de Abreu, Anna Clara Gregório Có, José Roberto Vasconcelos de Podestá, Melissa de Freitas Cordeiro-Silva, Sônia Alves Gouvea, Isabella Bittencourt do Valle, Sandra Lúcia Ventorin von Zeidler

**Affiliations:** 1 UFES, Department of Pathology, Health Sciences Center, Maruípe, 29040-090, Vitória, ES, Brazil; 2 UFES, Health Sciences Center, Maruípe, 29040-090, Vitória, ES, Brazil; 3 Hospital Santa Rita de Cassia, Prevention and Early Detection of Oral Cancer Program, Division of Head and Neck Surgery, Maruípe, 29040-091, Vitoria, ES, Brazil; 4 UFES, Center for Molecular and Human Genetics, Department of Biological Sciences, Center for Human and Natural Sciences - Maruípe, 29040-090, Vitoria, ES, Brazil; 5 UFES, Department of Physiological Sciences, Health Sciences Center - Maruípe, 29040-090, Vitoria, ES, Brazil

## Background

The oral squamous cell carcinoma (OSCC) is a malignant neoplasm originating from the epithelial lining of the oral cavity, accounting for about 95% of malignant lesions in this region. In Brazil, the oral cavity is the fifth location of the highest incidence of cancer in men and the seventh in women. In 2012, Brazil was estimated at 9,990 new cases of oral cancer in men and 4,180 in women [1]. The oral cavity and oropharynx present in the epithelium coating a wide variety of tissue patterns occupying very close areas. Thus, the study of squamous cell carcinoma of the oral cavity and oropharynx should consider the anatomical site, size and pattern of tumor infiltration as characteristics that may influence the biological behavior of the tumor [2]. This study aimed to evaluate the clinical and pathological aspects related to human papillomavirus (HPV) in squamous cell carcinoma of oral and oropharyngeal.

## Methods

Clinical data from 99 patients, from the Hospital Santa Rita and the Hospital Universitario Cassiano Antonio Moraes, diagnosed with squamous cell carcinoma of the oral cavity and oropharynx were obtained through interviews and analysis of records. The study population was categorized using criteria such as age, gender, history of smoking and alcohol and HPV infection. The clinical parameters of the location of the primary lesion, tumor size (T), regional lymph node involvement (N) and tumor invasion pattern were obtained by physical examination and pathology.

## Results and conclusions

The study population had an age range 30-93 years (mean 57.6 years), 80.8% of cases were male. Tobacco consumption was observed in 84.8% of patients, and alcohol consumption in 77.7%. High risk HPV types was detected in 4.04% of cases. Using the TNM system, it was observed that 27.27% of the cases had stage I and II and it were considered early stage, while 65.65% were in an advanced stage (stages III and IV). Most of the tumors had size T3 and T4 (57.57%). The most affected anatomical site was the oral cavity (83.83%), followed by the oropharynx (16.17%).

The tongue was the anatomic site more affected, fact that are compatible with the data found in the literature [3-5], which can also be related to a higher incidence of lymph node metastasis. These data reveal late diagnosis. There was no association between HPV infection and clinical aspects analyzed.

Low frequency of HPV infection can be justified by the majority of cases presenting primary lesion in the oral cavity and not in oropharynx, where frequency has been higher. However, in cases where human papillomavirus infection has been detected, greater impairment was observed with stages III and IV and primary tumor size T corresponding to T4, which means invading tumor tissues. In addition, many patients were heavy smoking and drinking, which are risk factors that increase the mutation of cells and may contribute to a worse prognosis.
